# What Makes a pDC: Recent Advances in Understanding Plasmacytoid DC Development and Heterogeneity

**DOI:** 10.3389/fimmu.2019.01222

**Published:** 2019-05-29

**Authors:** Andrea Musumeci, Konstantin Lutz, Elena Winheim, Anne Barbara Krug

**Affiliations:** Institute for Immunology, Biomedical Center, Ludwig-Maximilian-University, Munich, Germany

**Keywords:** plasmacytoid dendritic cells, hematopoiesis, dendritic cell development, DC progenitor, plasticity, heterogeneity

## Abstract

Dendritic cells (DCs) are professional antigen presenting cells (APCs) that originate in the bone marrow and are continuously replenished from hematopoietic progenitor cells. Conventional DCs (cDCs) and plasmacytoid DCs (pDCs) are distinguished by morphology and function, and can be easily discriminated by surface marker expression, both in mouse and man. Classification of DCs based on their ontology takes into account their origin as well as their requirements for transcription factor (TF) expression. cDCs and pDCs of myeloid origin differentiate from a common DC progenitor (CDP) through committed pre-DC stages. pDCs have also been shown to originate from a lymphoid progenitor derived IL-7R^+^ FLT3^+^ precursor population containing cells with pDC or B cell potential. Technological advancements in recent years have allowed unprecedented resolution in the analysis of cell states, down to the single cell level, providing valuable information on the commitment, and dynamics of differentiation of all DC subsets. However, the heterogeneity and functional diversification of pDCs still raises the question whether different ontogenies generate restricted pDC subsets, or fully differentiated pDCs retain plasticity in response to challenges. The emergence of novel techniques for the integration of high-resolution data in individual cells promises interesting discoveries regarding DC development and plasticity in the near future.

## Introduction

Plasmacytoid dendritic cells (pDCs) and two major subsets of conventional dendritic cells (cDC1 and cDC2) have been identified in mice and humans as well as other mammalian species including non-human primates and pigs, with high similarities between species (1–3). cDC subsets recognize both extracellular and intracellular pathogens, efficiently process and present exogenous antigens to naive CD4^+^ and CD8^+^ T cells and elicit effective adaptive immunity. pDCs are highly effective in sensing intracellular viral or self DNA and RNA mainly via Toll-like receptors (TLRs) and rapidly producing large amounts of type I and III interferons (IFNs) (4). Thus, they play an important role in antiviral immunity and systemic autoimmunity (5–8). pDCs are distinguished from cDC subsets by expression of surface markers CD45R (B220), CD45RA, Ly-6C, Siglec-H, and BST2 (CD317) in the mouse and CD303 (BDCA2), CD304 (BDCA4), CD123 (IL-3R), and CD45RA in humans.

**Figure 1:**
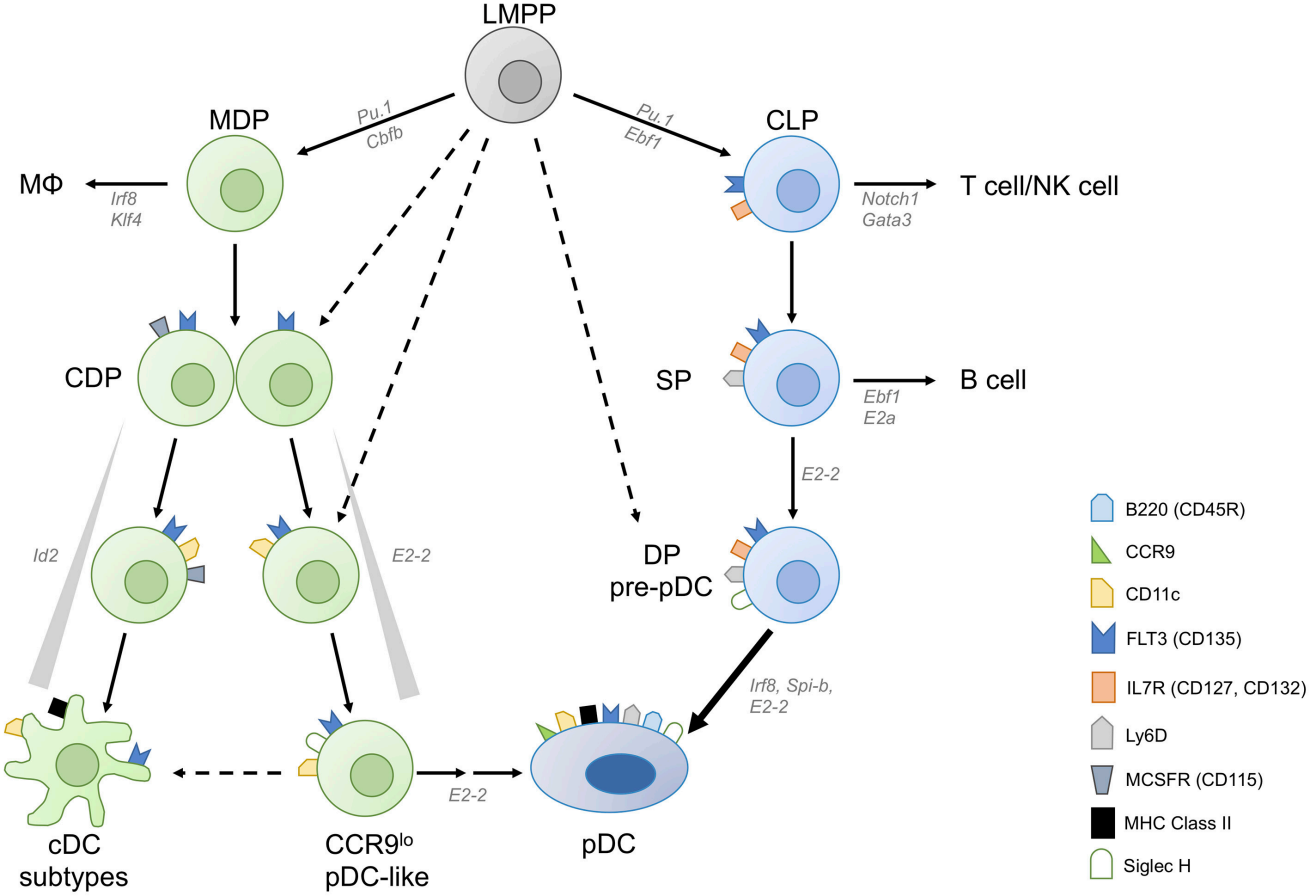
Converging plasmacytoid dendritic cell differentiation pathways. Plasmacytoid dendritic cells (pDCs) can be derived from both myeloid and lymphoid progenitors. Common DC progenitors (CDPs) arise from lymphoid primed multipotent progenitors (LMPPs) either directly or via macrophage-DC progenitors (MDPs). CDPs contain precursor cells committed to conventional DC (cDC) and plasmacytoid DC fates, and M-CSFR^−^ CDPs have higher pDC potential than M-CSFR^+^ CDPs. A fraction of CDPs can give rise to CCR9^low^ pDC-like precursor cells and then CCR9^high^ pDCs in an E2-2 dependent manner. pDC-like cells retain the potential to differentiate into cDCs as well as CCR9^high^ mature pDCs. Inhibitor of DNA binding 2 (Id2), which inhibits E2-2 activity, needs to be suppressed to allow pDC differentiation. pDCs are also generated via the lymphoid pathway, from IL-7R^+^ lymphoid progenitors (LPs) which give rise to Ly-6D single positive (SP) LP and subsequently to Ly-6D Siglec-H double positive (DP) pre-pDC, terminally committed to the pDC fate.

DC subpopulations originate from proliferating progenitor cells in the bone marrow (BM) and require fms-like tyrosine kinase 3 ligand (FLT3L)–FLT3 interaction for their development. Lin^−^ FLT3^+^ c-Kit^low/int^ M-CSFR^+^ murine BM cells, so called common DC progenitors (CDP), which are derived from the myeloid macrophage DC progenitors (MDP) or lymphoid primed multipotent progenitors (LMPP), were shown to be DC-committed and to generate pDCs, cDC1 and cDC2 [Figure 1, (9, 10)]. Clonal assays and subsequent single cell transcriptome and imaging analyses demonstrated that the majority of CDPs are already pre-committed to pDC or cDC subsets (9–13). This is also the case for the pre-cDCs, which already contain pre-cDC1, and pre-cDC2 (13, 14). In contrast, pDCs are also produced from a lymphoid progenitor (LP) (15) in the steady state whereas this happens for cDCs only in situation of cDC ablation (16).

DC subpopulations can be defined by their ontogeny and by the requirement of specific transcription factors (TF) for their development. pDCs require high-level expression of IRF-8, TCF-4 (also known as E2-2) and BCL-11A for their development, functional specification and maintenance (17–21). Expression of DNA-binding protein inhibitor ID-2, which prevents the activity of the major pDC TF E2-2, needs to be suppressed to allow the generation of pDCs from CDPs (22, 23). On the other hand, the major cDC branches can be distinguished by distinct requirements for IRF-8 (for cDC1) and IRF-4 (for cDC2) (14, 24–27).

DC subpopulations are also distinguished by a high degree of functional specialization (28). While cDC1 efficiently cross-present antigens to CD8^+^ T cells (27, 29, 30) and produce high levels of IL-12p70, thus promoting cytotoxic T cells and Th1 cells (31, 32), cDC2 are superior in presenting antigens on MHC class II, supporting Th1, Th2, and Th17 polarization (26, 27, 33). pDCs participate in the first line of defense against viral infections by acting as innate effector cells, which initiate IFN-induced antiviral responses in adjacent cells and recruit cytotoxic NK cells (5). Resting pDCs are weak antigen presenting cells and in contrast to cDCs do not prime naïve T cells. After activation, pDCs can acquire the capacity to present antigens and activate T cells directly. Their ability to prime T cells, thus performing truly like DCs, is debated and complicated by the finding that pDC-like cells, which were shown to be related to cDCs (13, 15, 34, 35) have been included in the pDC population in many functional studies, as discussed below. By producing cytokines and chemokines activated pDCs modulate T cell responses elicited by cDCs (5). During viral infection pDCs were shown to cooperate with cDC1 in lymph nodes, promoting their maturation and cross-presentation activity to induce antiviral CD8^+^ T cells (36). But there is also evidence for a role of pDCs in the induction of immune tolerance by generation of hyporesponsive and regulatory T cells (37–39).

Recent technological developments have allowed unprecedented resolution, down to the single cell level, in the analysis of cell transcriptomes as well as in *in vivo* lineage tracing, overcoming the limitations of discrimination based solely on surface markers (40–44). The characterization of transcriptional profiles of individual cells (13, 42, 45) and more recently the integrated analysis of single cell transcriptome and chromatin accessibility (46) has revealed unexpected heterogeneity and signs of very early lineage priming of individual hematopoietic BM progenitor cells, which were previously considered multi- or oligopotent. For example, single cell barcoding and tracing showed that DC and even pDC commitment can already be imprinted in early LMPP and at the HSPC stage (12, 41, 47). cDC subtype specification was detected already at the CDP and pre-cDC stage of development (12–14). In some instances, these analyses led to the definition of more stringent surface marker combinations that allow the discrimination of largely committed progenitor cells within the “oligopotent” population (13, 15).

Combining CRISPR/Cas9-based genomic perturbation with transcriptome profiling in the same cells revealed differentiation trajectories and regulatory networks during hematopoiesis (40, 48). Integration of clonal labeling and lineage tracing experiments and single cell time-lapse imaging experiments may lead to a better understanding of immune cell differentiation dynamics and regulation in the future (11, 40, 43, 49).

## Plasmacytoid Dendritic Cell Development From Myeloid and Lymphoid Progenitors

Early works indicated that DCs can be derived from both FLT3^+^ CMP and CLP (50, 51). Competitive *in vivo* transfer experiments with CMPs and CLPs showed that pDCs can also be generated from both, but are mainly of “myeloid” origin (52). Subsequent studies indicated that CMP and CLP-derived pDCs differ in their ability to produce type I IFN and to stimulate T cells (53, 54). Interestingly, a significant proportion of pDCs expresses recombination activation genes (*Rag1*/*Rag2*) and undergoes immunoglobulin D_H_-J_H_ rearrangement indicating a “lymphoid” past. But the expression of Rag genes and detection of Ig rearrangements in pDCs derived from both CMP and CLP suggested that these are by-products of a “lymphoid” transcriptional program expressed only transiently in the pDC lineage (55, 56). However, the issue was revisited by Sathe et al. who found that RAG1 expression and Ig rearrangement are mainly found in CLP-derived pDCs (54). pDC generation from CLPs but not CDPs required constitutive type I IFN signals for upregulation of FLT3, suggesting differential requirements for instructive cytokines for the two developmental pathways (57). After the discovery that myeloid progenitor derived CDPs generate both cDCs and pDCs, research mostly focused on the branching of pDC and cDC development.

We found that CCR9^low^ pDC-like precursor cells (CD11c+ Siglec H+ BST2+ B220lo/hi), which express lower levels of E2-2 and higher levels of Id2 than pDCs, can be generated from murine CDPs and these can give rise to CCR9^high^ pDCs as well as cDCs [Figure 1, (11, 58, 59)]. The CCR9^low^ pDC-like precursor population in the BM contains only a small fraction of proliferating cells indicating heterogeneity within this population regarding differentiation stage (58). It remains to be determined if this population, which can also be detected in lymphoid organs at low frequency contains differentiated cells with plasticity to develop into pDCs and cDCs or precursors with dual potential or both. Interestingly, pDC-like cells with a similar phenotype accumulated in the BM of Mtg16-deficient mice, which failed to downregulate Id2 expression, thereby blocking the activity of E2-2 and further pDC differentiation (60). In addition, Zeb2 has been identified as an important regulator of Id2 expression, which allows pDC development from CDPs by suppressing the alternative cDC1 fate at a common precursor stage (22, 23). More recently Etv6 was shown to cooperate with IRF8 to refine cDC1-specific gene expression and repress the pDC gene expression signature indicating the close relationship between cDC1 and pDCs (61). Siglec-H, a canonical marker distinguishing mature pDCs from cDCs, is expressed at very early stages of differentiation, but does not denote a plasmacytoid commitment. Within the CDP and the pre-DC fraction in the BM, Siglec-H^+^ cells expressing TF Zbtb46 are exclusively committed to cDCs (62) and were shown to contain precursors committed to cDC1 and cDC2 (13, 14). Similarly, Siglec-H^+^ Ly-6C^+^ cells in the pre-DC compartment (defined as Lin^−^ CD135^+^ CD11c^+^ MHCII^−^ CD172α^−^) were shown to give rise to both subsets of cDCs, whereas Siglec-H^+^ Ly-6C^−^ pre-DCs gave rise to cDC subsets and pDCs (13). Using the single cell imaging and tracking method we could show that CDP progeny transit through a CD11c^+^ CCR9^low^ Siglec-H^+^ pDC-like stage during their development into CCR9^high^ pDCs (11). The CDP-derived pDC-committed precursor, which must be present within this population, is still a missing link. M-CSFR^+^ CDPs give rise to pDCs, however their output is rather low. Interestingly, Onai et al. found that the pDC potential was higher in the M-CSFR^−^ E2-2^+^ fraction of CDPs in murine BM (12, 63). They also demonstrated that E2-2^high^ cells within M-CSFR^−^ IL-7R^−^ CDPs gave rise exclusively to pDCs in spleen and lymph nodes, but also to cDCs in the small intestine, showing the plasticity of this pDC-primed CDP subset or its progeny in the local tissue environment (63).

More recently Rodrigues et al. found that FLT3^+^ IL-7R (CD127)^+^ CD117^lo/int^ lymphoid progenitor (LP) cells in murine BM, which differ from CDPs only by expression of IL-7R and lack of M-CSFR expression, have a 5-fold higher output of pDCs compared to CDPs (15). Within this LP pool, three subpopulations were distinguished by diverse expression of Siglec-H and Ly-6D. Of these, only the Siglec-H Ly-6D double positive (DP) population had exclusive pDC potential, while the Ly-6D single positive (SP) population generated both B cells and pDCs, congruent with the results of a recently published computational fate mapping analysis of single cell RNAseq data (64). Further analysis showed the SP population to contain cells committed either to B cell or to pDC differentiation. The model proposed by Rodrigues et al. suggests that IL-7R^+^ Siglec-H and Ly-6D DN LPs proceed to upregulate Ly-6D (SP) and, under the influence of lineage defining TFs IRF8 and EBF1 induced by FLT3L and IL-7 respectively, proceed either to the pDC lineage or towards B cells (Figure 1). Interestingly, mice lacking Zeb2 in CD11c^+^ cells were shown to have a severe defect in pDC numbers, which was attributed to failed repression of Id2 leading to diversion of precursors to cDC1 (22, 23). Since a substantial proportion of pDCs was shown to be derived from the LP which lacks cDC potential in the steady state (65), it remains to be investigated if the transcriptional repressor Zeb2 is also involved in suppressing alternative cell fates in the LP.

Functionally, the IL-7R^+^ DP cells described by Rodriguez et al. as pDC precursors can be considered immature progenitors, as they do not yet express genes important for pDC function (such as *Irf7* and *Spib*) and require further cell divisions to generate mature pDCs (15). In contrast to the CDP-derived CD11c^+^ Siglec-H^+^ CCR9^low^ pDC-like precursors, the IL-7R^+^ DP cells lack CD11c and B220 expression and fail to produce type I IFNs in response to TLR9 stimulation by CpG-A, a hallmark of the pDC-lineage, but acquire this capacity after culture with FLT3L (15).

IL-7R^+^ Siglec-H^+^ Ly-6D^+^ pDC-committed precursors make a substantial contribution to the pool of differentiated pDCs. Thus, pDC generation seems to be regulated by the cell fate decision between pDC and cDC1, but also by the pDC versus B cell dichotomy. The contribution of the two pathways to pDC generation under conditions of inflammation or infection and the functional consequences of the distinct ontogeny of pDCs remain to be investigated.

## Heterogeneity of pDCs and pDC-Like Cells in Murine Lymphoid Organs

Different subsets of pDCs have been identified in the BM, mostly differing in their degree of differentiation and their capacity to produce type I IFNs or pro-inflammatory cytokines (4, 66). Markers such as CCR9, SCA-1, CD9, and Ly-49Q, which are expressed by the majority of peripheral mouse pDCs, can be used to discriminate these subsets (59, 67, 68). More recently, single cell RNAseq analysis confirmed the presence of two subsets within Lin^−^ CD11c^+^ BST2^+^ Siglec-H^+^ cells in spleen and BM (15). The “pDC-like cells” described in this paper express several genes characteristic of cDCs and other myeloid cells (including *Zbtb46*) but lack or express low levels of *Ccr9, Ly6d*, and *Dntt*. By gene expression profile and surface phenotype (lower levels of Siglec-H, BST2, MHCII, higher levels of CD11c, Ly-6C, and CX3CR1 compared to pDCs) they greatly resemble the CCR9^low^ MHCII^low^ CX3CR1^+^ pDC-like precursors described previously in BM (58, 59) and are a subset of those. Interestingly, Rodrigues et al. also found that the minor subset of pDC-like cells (defined as Zbtb46-eGFP^+^ Siglec-H^int^ BST2^+^), responded with IFN-α production to CpG-A and showed better antigen processing and presenting ability than “regular” pDCs. It was also previously shown that IFN-β production in the spleen is limited to a small subset of CD9^−^ cells within the CCR9^+^ mature pDC population in murine spleen (69).

These works suggest the existence of minor subsets of pDCs in peripheral organs, differing in the extent of IFN-I production and the capacity of antigen processing and presentation. Considering that these subsets identified by differential expression of surface markers are largely overlapping and often very rare, it remains unclear whether the functional differences observed are due to functional specialization or are the result of lineage imprinting, or whether they are simply sequential stages of pDC differentiation leading to the mature pDC.

## Revisiting the Definition of Human pDCs

The pDC-like cells described in the mouse which express pDC markers and TFs, but rapidly give rise to cDCs and behave like cDCs in antigen presentation assays greatly resemble the subset of CD123^+^ CD45RA^+^ CD33^+^ CX3CR1^+^ pre-DCs recently identified in human blood (35) and the AXL^+^ SIGLEC6^+^ human blood DC subset (AS-DC) described by Villani et al. (34). These “pDC-like cells,” which are hidden in the pDC population as defined by surface marker expression (Lin^−^ HLA-DR^+^ CD123^+^ CD45RA^+^ CD303^+^), are functionally distinct from pDC in that they do not produce type I IFN in response to TLR7 and 9 stimulation. In that respect they are different from the Zbtb46^+^ Siglec-H^+^ pDC-like cells found in murine spleen. As to their classification as precursors of cDCs, it is based mainly on the observation that the pre-DCs acquire cDC phenotype and function in culture (35). The human pre-DC population contains pre-cDC1 and pre-cDC2 (35, 70). However, these cells are not proliferating in the steady state and appear to be functionally mature and could therefore actively participate in immune responses (34, 35). Cells in human blood, BM and tonsil defined as a CD2^+^ CD5^+^ (and CD81^+^) subpopulation of human pDCs were studied previously and were found to produce IL-12 but not IFN-α and to stimulate naïve CD4^+^ T cells (71–74). This population is largely overlapping with the recently described pre-DC and AS-DC (34, 35). It is currently not resolved to which extent cytokine responses and T cell activation capacity attributed to human pDCs in earlier studies were influenced by contamination by cDC precursors, especially because most studies were performed with pDCs that had been stimulated e.g., with IL-3, CD40L or viruses (75–77). It was shown recently that human blood pDCs diversify into functionally distinct and stable subsets after activation by influenza virus or CpG even after prior exclusion of contaminating pre-DCs demonstrating great functional plasticity of this cell type (78). In the light of these recent findings the functional properties of *bona fide* pDCs in innate and adaptive immune responses need to be reexamined.

## Future Perspectives

Technological advances including single cell transcriptome, epigenome, and mass cytometry analyses as well as single cell tracking methods have revealed that development and functional specification of DC subpopulations is much more complex than anticipated. Several questions regarding pDC development and functional plasticity remain unanswered. It would be important to address the contribution of the CDP and LP to pDCs during infections or inflammation and to clarify if the developmental history of pDCs is really relevant for their function. Furthermore, it is unclear at this point, which functions ascribed to human pDCs are mediated by *bona fide* pDCs and which are mediated by the contaminating pre-DCs. This is especially important for developing pDC-targeted or adoptive transfer therapies for induction of immunity or tolerance. Similarly, the functional diversification of pDCs after activation and also the phenomenon of pDC exhaustion during chronic infection (79) are important topics for further study. An exciting area of research is the correlation of gene expression with chromatin accessibility and epigenetic modifications on the single cell level and the integration of all this data (80), which will allow to unravel the transcriptional regulation of cell fate decisions leading to pDC development and functional diversification. Combined with CRISPR/Cas9-based genetic screening and functional assays these new single cell analysis methods will lead to a thorough understanding of development, plasticity and function of DC subpopulations with implications for DC targeted therapy.

## Author Contributions

All authors listed have made a substantial, direct and intellectual contribution to the work, and approved it for publication.

### Conflict of Interest Statement

The authors declare that the research was conducted in the absence of any commercial or financial relationships that could be construed as a potential conflict of interest.

## References

[1] Auray G Keller I Python S Gerber M Bruggmann R Ruggli N. Characterization and transcriptomic analysis of porcine blood conventional and plasmacytoid dendritic cells reveals striking species-specific differences. J Immunol. (2016) 197:4791–806. 10.4049/jimmunol.1600672 27837108

[2] Guilliams M Dutertre CA Scott CL McGovern N Sichien D Chakarov S. Unsupervised high-dimensional analysis aligns dendritic cells across tissues and species. Immunity. (2016) 45:669–84. 10.1016/j.immuni.2016.08.015 27637149 PMC5040826

[3] Heidkamp GF Sander J Lehmann CHK Heger L Eissing N Baranska A Human lymphoid organ dendritic cell identity is predominantly dictated by ontogeny, not tissue microenvironment. Sci Immunol. (2016) 1:eaai7677 10.1126/sciimmunol.aai7677 28783692

[4] Swiecki M Colonna M. The multifaceted biology of plasmacytoid dendritic cells. Nat Rev Immunol. (2015) 15:471–85. 10.1038/nri3865 26160613 PMC4808588

[5] Swiecki M Gilfillan S Vermi W Wang Y Colonna M. Plasmacytoid dendritic cell ablation impacts early interferon responses and antiviral NK and CD8(+) T cell accrual. Immunity. (2010) 33:955–66. 10.1016/j.immuni.2010.11.020 21130004 PMC3588567

[6] Rowland SL Riggs JM Gilfillan S Bugatti M Vermi W Kolbeck R. Early, transient depletion of plasmacytoid dendritic cells ameliorates autoimmunity in a lupus model. J Exp Med. (2014) 211:1977–91. 10.1084/jem.20132620 25180065 PMC4172228

[7] Sisirak V Ganguly D Lewis KL Couillault C Tanaka L Bolland S. Genetic evidence for the role of plasmacytoid dendritic cells in systemic lupus erythematosus. J Exp Med. (2014) 211:1969–76. 10.1084/jem.20132522 25180061 PMC4172218

[8] Ah Kioon MD Tripodo C Fernandez D Kirou KA Spiera RF Crow MK. Plasmacytoid dendritic cells promote systemic sclerosis with a key role for TLR8. Sci Transl Med. (2018) 10:eaam8458. 10.1126/scitranslmed.aam8458 29321259 PMC9865429

[9] Naik SH Sathe P Park H-Y Metcalf D Proietto AI Dakic A. Development of plasmacytoid and conventional dendritic cell subtypes from single precursor cells derived *in vitro* and *in vivo*. Nat Immunol. (2007) 8:1217–26. 10.1038/ni1522 17922015

[10] Onai N Obata-Onai A Schmid MA Ohteki T Jarrossay D Manz MG. Identification of clonogenic common Flt3+M-CSFR+ plasmacytoid and conventional dendritic cell progenitors in mouse bone marrow. Nat Immunol. (2007) 8:1207–16. 10.1038/ni1518 17922016

[11] Dursun E Endele M Musumeci A Failmezger H Wang S-H Tresch A. Continuous single cell imaging reveals sequential steps of plasmacytoid dendritic cell development from common dendritic cell progenitors. Scient Rep. (2016) 6:37462. 10.1038/srep37462 27892478 PMC5124969

[12] Onai N Kurabayashi K Hosoi-Amaike M Toyama-Sorimachi N Matsushima K Inaba K. A clonogenic progenitor with prominent plasmacytoid dendritic cell developmental potential. Immunity. (2013) 38:943–57. 10.1016/j.immuni.2013.04.006 23623382

[13] Schlitzer A Sivakamasundari V Chen J Sumatoh HRB Schreuder J Lum J. Identification of cDC1- and cDC2-committed DC progenitors reveals early lineage priming at the common DC progenitor stage in the bone marrow. Nat Immunol. (2015) 16:718–28. 10.1038/ni.3200 26054720

[14] Grajales-Reyes GE Iwata A Albring J Wu X Tussiwand R KC W. Batf3 maintains autoactivation of Irf8 for commitment of a CD8α(+) conventional DC clonogenic progenitor. Nat Immunol. (2015) 16:708–17. 10.1038/ni.3197 26054719 PMC4507574

[15] Rodrigues PF Alberti-Servera L Eremin A Grajales-Reyes GE Ivanek R Tussiwand R Distinct progenitor lineages contribute to the heterogeneity of plasmacytoid dendritic cells. Nat Immunol. (2018) 392:245 10.1038/s41590-018-0136-9 PMC7614340 29925996

[16] Salvermoser J van Blijswijk J Papaioannou NE Rambichler S Pasztoi M Pakalniskyte D. Clec9a-mediated ablation of conventional dendritic cells suggests a lymphoid path to generating dendritic cells *in vivo*. Front Immunol. (2018) 9:699. 10.3389/fimmu.2018.00699 29713321 PMC5911463

[17] Ippolito GC Dekker JD Wang YH Lee BK Shaffer AL III Lin J. Dendritic cell fate is determined by BCL11A. Proc Natl Acad Sci USA. (2014) 111:E998–1006. 10.1073/pnas.1319228111 24591644 PMC3964079

[18] Cisse B Caton ML Lehner M Maeda T Scheu S Locksley R. Transcription factor E2–2 is an essential and specific regulator of plasmacytoid dendritic cell development. Cell. (2008) 135:37–48. 10.1016/j.cell.2008.09.016 18854153 PMC2631034

[19] Ghosh HS Cisse B Bunin A Lewis KL Reizis B. Continuous expression of the transcription factor E2–2 maintains the cell fate of mature plasmacytoid dendritic cells. Immunity. (2010) 33:905–16. 10.1016/j.immuni.2010.11.023 21145760 PMC3010277

[20] Grajkowska LT Ceribelli M Lau CM Warren ME Tiniakou I Nakandakari Higa S. Isoform-specific expression and feedback regulation of E protein TCF4 control dendritic cell lineage specification. Immunity. (2017) 46:65–77. 10.1016/j.immuni.2016.11.006 27986456 PMC5243153

[21] Sichien D Scott CL Martens L Vanderkerken M Van Gassen S Plantinga M. IRF8 transcription factor controls survival and function of terminally differentiated conventional and plasmacytoid dendritic cells, respectively. Immunity. (2016) 45:626–40. 10.1016/j.immuni.2016.08.013 27637148

[22] Scott CL Soen B Martens L Skrypek N Saelens W Taminau J. The transcription factor Zeb2 regulates development of conventional and plasmacytoid DCs by repressing Id2. J Exp Med. (2016) 213:897–911. 10.1084/jem.20151715 27185854 PMC4886362

[23] Wu X Briseño CG Grajales-Reyes GE Haldar M Iwata A Kretzer NM. Transcription factor Zeb2 regulates commitment to plasmacytoid dendritic cell and monocyte fate. Proc Natl Acad Sci USA. (2016) 113:14775–80. 10.1073/pnas.1611408114 27930303 PMC5187668

[24] Murphy TL Grajales-Reyes GE Wu X Tussiwand R Briseño CG Iwata A. Transcriptional control of dendritic cell development. Annu Rev Immunol. (2016) 34:93–119. 10.1146/annurev-immunol-032713-120204 26735697 PMC5135011

[25] Miller JC Brown BD Gautier EL Jojic V Cohain A Pandey G. Deciphering the transcriptional network of the dendritic cell lineage. Nat Immunol. (2012) 13:888–99. 10.1038/ni.2370 22797772 PMC3985403

[26] Schlitzer A McGovern N Teo P Zelante T Atarashi K Low D. IRF4 transcription factor-dependent CD11b+ dendritic cells in human and mouse control mucosal IL-17 cytokine responses. Immunity. (2013) 38:970–83. 10.1016/j.immuni.2013.04.011 23706669 PMC3666057

[27] Williams JW Tjota MY Clay BS Vander Lugt B Bandukwala HS Hrusch CL. Transcription factor IRF4 drives dendritic cells to promote Th2 differentiation. Nat Commun. (2013) 4:2990. 10.1038/ncomms3990 24356538 PMC4003872

[28] Dress RJ Wong AY Ginhoux F. Homeostatic control of dendritic cell numbers and differentiation. Immunol Cell Biol. (2018) 96:463–76. 10.1111/imcb.12028 29473216

[29] den Haan JMM Lehar SM Bevan MJ Cd8+but not Cd8–dendritic cells cross-prime cytotoxic T cells *in vivo*. J Exp Med. (2000) 192:1685–96. 10.1084/jem.192.12.1685 11120766 PMC2213493

[30] Segura E Amigorena S. Cross-presentation by human dendritic cell subsets. Immunol Lett. (2014) 158:73–8. 10.1016/j.imlet.2013.12.001 24333339

[31] Nizzoli G Krietsch J Weick A Steinfelder S Facciotti F Gruarin P. Human CD1c+ dendritic cells secrete high levels of IL-12 and potently prime cytotoxic T-cell responses. Blood. (2013) 122:932–42. 10.1182/blood-2013-04-495424 23794066

[32] Shortman K Heath WR. The CD8+ dendritic cell subset. Immunol Rev. (2010) 234:18–31. 10.1111/j.0105-2896.2009.00870.x 20193009

[33] Dudziak D Kamphorst AO Heidkamp GF Buchholz VR Trumpfheller C Yamazaki S. Differential antigen processing by dendritic cell subsets *in vivo*. Science. (2007) 315:107–11. 10.1126/science.1136080 17204652

[34] Villani AC Satija R Reynolds G Sarkizova S Shekhar K Fletcher J. Single-cell RNA-seq reveals new types of human blood dendritic cells, monocytes, and progenitors. Science. (2017) 356:eaah4573. 10.1126/science.aah4573 28428369 PMC5775029

[35] See P Dutertre C-A Chen J Günther P McGovern N Irac SE. Mapping the human DC lineage through the integration of high-dimensional techniques. Science. (2017) 356:eaag3009. 10.1126/science.aag3009 28473638 PMC7611082

[36] Brewitz A Eickhoff S Dahling S Quast T Bedoui S Kroczek RA. CD8(+) T cells orchestrate pDC-XCR1(+) dendritic cell spatial and functional cooperativity to optimize priming. Immunity. (2017) 46:205–19. 10.1016/j.immuni.2017.01.003 28190711 PMC5362251

[37] Loschko J Heink S Hackl D Dudziak D Reindl W Korn T. Antigen targeting to plasmacytoid dendritic cells via Siglec-H inhibits Th cell-dependent autoimmunity. J Immunol. (2011) 187:6346–56. 10.4049/jimmunol.1102307 22079988

[38] Irla M Kupfer N Suter T Lissilaa R Benkhoucha M Skupsky J. MHC class II-restricted antigen presentation by plasmacytoid dendritic cells inhibits T cell-mediated autoimmunity. J Exp Med. (2010) 207:1891–905. 10.1084/jem.20092627 20696698 PMC2931160

[39] Hadeiba H Lahl K Edalati A Oderup C Habtezion A Pachynski R. Plasmacytoid dendritic cells transport peripheral antigens to the thymus to promote central tolerance. Immunity. (2012) 36:438–50. 10.1016/j.immuni.2012.01.017 22444632 PMC3315699

[40] Giladi A Paul F Herzog Y Lubling Y Weiner A Yofe I. Single-cell characterization of haematopoietic progenitors and their trajectories in homeostasis and perturbed haematopoiesis. Nat Cell Biol. (2018) 20:836–46. 10.1038/s41556-018-0121-4 29915358

[41] Naik SH Perie L Swart E Gerlach C van Rooij N de Boer RJ. Diverse and heritable lineage imprinting of early haematopoietic progenitors. Nature. (2013) 496:229–32. 10.1038/nature12013 23552896

[42] Paul F Arkin Ya Giladi A Jaitin DA Kenigsberg E Keren-Shaul H. Transcriptional heterogeneity and lineage commitment in myeloid progenitors. Cell. (2015) 163:1663–77. 10.1016/j.cell.2015.11.013 26627738

[43] Pei W Feyerabend TB Rossler J Wang X Postrach D Busch K. Polylox barcoding reveals haematopoietic stem cell fates realized *in vivo*. Nature. (2017) 548:456–60. 10.1038/nature23653 28813413 PMC5905670

[44] Schraml BU van Blijswijk J Zelenay S Whitney PG Filby A Acton SE. Genetic tracing via DNGR-1 expression history defines dendritic cells as a hematopoietic lineage. Cell. (2013) 154:843–58. 10.1016/j.cell.2013.07.014 23953115

[45] Perie L Duffy KR Kok L de Boer RJ Schumacher TN. The branching point in erythro-myeloid differentiation. Cell. (2015) 163:1655–62. 10.1016/j.cell.2015.11.059 26687356

[46] Buenrostro JD Corces MR Lareau CA Wu B Schep AN Aryee MJ. Integrated single-cell analysis maps the continuous regulatory landscape of human hematopoietic differentiation. Cell. (2018) 173:1535–48 e16. 10.1016/j.cell.2018.03.074 29706549 PMC5989727

[47] Lin DS Kan A Gao J Crampin EJ Hodgkin PD Naik SH. DiSNE movie visualization and assessment of clonal kinetics reveal multiple trajectories of dendritic cell development. Cell Rep. (2018) 22:2557–66. 10.1016/j.celrep.2018.02.046 29514085

[48] Jaitin DA Weiner A Yofe I Lara-Astiaso D Keren-Shaul H David E. Dissecting immune circuits by linking CRISPR-pooled screens with single-cell RNA-Seq. Cell. (2016) 167:1883–96 e15. 10.1016/j.cell.2016.11.039 27984734

[49] Hoppe PS Coutu DL Schroeder T. Single-cell technologies sharpen up mammalian stem cell research. Nat Cell Biol. (2014) 16:919–27. 10.1038/ncb3042 25271480

[50] Karsunky H Merad M Cozzio A Weissman IL Manz MG. Flt3 ligand regulates dendritic cell development from Flt3+ lymphoid and myeloid-committed progenitors to Flt3+ dendritic cells *in vivo*. J Exp Med. (2003) 198:305–13. 10.1084/jem.20030323 12874263 PMC2194067

[51] Manz MG Traver D Miyamoto T Weissman IL Akashi K. Dendritic cell potentials of early lymphoid and myeloid progenitors. Blood. (2001) 97:3333–41. 10.1182/blood.V97.11.3333 11369621

[52] Karsunky H Merad M Mende I Manz MG Engleman EG Weissman IL. Developmental origin of interferon-alpha-producing dendritic cells from hematopoietic precursors. Exp Hematol. (2005) 33:173–81. 10.1016/j.exphem.2004.10.010 15676211

[53] Yang G-X Lian Z-X Kikuchi K Moritoki Y Ansari AA Liu Y-J. Plasmacytoid dendritic cells of different origins have distinct characteristics and function: studies of lymphoid progenitors versus myeloid progenitors. J Immunol. (2005) 175:7281–7. 10.4049/jimmunol.175.11.7281 16301633

[54] Sathe P Vremec D Wu L Corcoran L Shortman K. Convergent differentiation: myeloid and lymphoid pathways to murine plasmacytoid dendritic cells. Blood. (2013) 121:11–9. 10.1182/blood-2012-02-413336 23053574

[55] Pelayo R Hirose J Huang J Garrett KP Delogu A Busslinger M. Derivation of 2 categories of plasmacytoid dendritic cells in murine bone marrow. Blood. (2005) 105:4407–15. 10.1182/blood-2004-07-2529 15728131 PMC1850236

[56] Shigematsu H Reizis B Iwasaki H Mizuno S-i Hu D Traver D. Plasmacytoid dendritic cells activate lymphoid-specific genetic programs irrespective of their cellular origin. Immunity. (2004) 21:43–53. 10.1016/j.immuni.2004.06.011 15345219

[57] Chen Y-L Chen T-T Pai L-M Wesoly J Bluyssen HAR Lee C-K. A type I IFN-Flt3 ligand axis augments plasmacytoid dendritic cell development from common lymphoid progenitors. J Exp Med. (2013) 210:2515–22. 10.1084/jem.20130536 24145513 PMC3832917

[58] Schlitzer A Heiseke AF Einwächter H Reindl W Schiemann M Manta C-P. Tissue-specific differentiation of a circulating CCR9- pDC-like common dendritic cell precursor. Blood. (2012) 119:6063–71. 10.1182/blood-2012-03-418400 22547585

[59] Schlitzer A Loschko J Mair K Vogelmann R Henkel L Einwächter H. Identification of CCR9- murine plasmacytoid DC precursors with plasticity to differentiate into conventional DCs. Blood. (2011) 117:6562–70. 10.1182/blood-2010-12-326678 21508410

[60] Ghosh HS Ceribelli M Matos I Lazarovici A Bussemaker HJ Lasorella A. ETO family protein Mtg16 regulates the balance of dendritic cell subsets by repressing Id2. J Exp Med. (2014) 211:1623–35. 10.1084/jem.20132121 24980046 PMC4113936

[61] Lau CM Tiniakou I Perez OA Kirkling ME Yap GS Hock H. Transcription factor Etv6 regulates functional differentiation of cross-presenting classical dendritic cells. J Exp Med. (2018) 215:2265–78. 10.1084/jem.20172323 30087163 PMC6122974

[62] Satpathy AT KC W Albring JC Edelson BT Kretzer NM Bhattacharya D. Zbtb46 expression distinguishes classical dendritic cells and their committed progenitors from other immune lineages. J Exp Med. (2012) 209:1135–52. 10.1084/jem.20120030 22615127 PMC3371733

[63] Onai N Asano J Kurosaki R Kuroda S Ohteki T. Flexible fate commitment of E2–2high common DC progenitors implies tuning in tissue microenvironments. Int Immunol. (2017) 29:443–56. 10.1093/intimm/dxx058 29106601

[64] Herman JS Sagar Grun D. FateID infers cell fate bias in multipotent progenitors from single-cell RNA-seq data. Nat Methods. (2018) 15:379–86. 10.1038/nmeth.4662 29630061

[65] Rodriguez-Fraticelli AE Wolock SL Weinreb CS Panero R Patel SH Jankovic M. Clonal analysis of lineage fate in native haematopoiesis. Nature. (2018) 553:212–6. 10.1038/nature25168 29323290 PMC5884107

[66] Macri C Pang ES Patton T O'Keeffe M. Dendritic cell subsets. Semin Cell Dev Biol. (2017) 84:11–21. 10.1016/j.semcdb.2017.12.009 29246859

[67] Niederquell M Kurig S Fischer JAA Tomiuk S Swiecki M Colonna M Sca-1 expression defines developmental stages of mouse pDCs that show functional heterogeneity in the endosomal but not lysosomal TLR9 response. Eur J Immunol. (2013) 43:2993–3005. 10.1002/eji.201343498 23922217

[68] Kamogawa-Schifter Y Ohkawa J Namiki S Arai N Arai K-I Liu Y. Ly49Q defines 2 pDC subsets in mice. Blood. (2005) 105:2787–92. 10.1182/blood-2004-09-3388 15598811

[69] Bauer J Dress RJ Schulze A Dresing P Ali S Deenen R. Cutting Edge: IFN-beta expression in the spleen is restricted to a subpopulation of plasmacytoid dendritic cells exhibiting a specific immune modulatory transcriptome signature. J Immunol. (2016) 196:4447–51. 10.4049/jimmunol.1500383 27183572

[70] Breton G Zheng S Valieris R Tojal da Silva I Satija R Nussenzweig MC. Human dendritic cells. (DCs) are derived from distinct circulating precursors that are precommitted to become CD1c+ or CD141+ DCs. J Exp Med. (2016) 213:2861–70. 10.1084/jem.20161135 27864467 PMC5154947

[71] Bryant C Fromm PD Kupresanin F Clark G Lee K Clarke C. A CD2 high-expressing stress-resistant human plasmacytoid dendritic-cell subset. Immunol Cell Biol. (2016) 94:447–57. 10.1038/icb.2015.116 26791160

[72] Matsui T Connolly JE Michnevitz M Chaussabel D Yu CI Glaser C. CD2 distinguishes two subsets of human plasmacytoid dendritic cells with distinct phenotype and functions. J Immunol. (2009) 182:6815–23. 10.4049/jimmunol.0802008 19454677 PMC2749454

[73] Zhang H Gregorio JD Iwahori T Zhang X Choi O Tolentino LL. A distinct subset of plasmacytoid dendritic cells induces activation and differentiation of B and T lymphocytes. Proc Natl Acad Sci USA. (2017) 114:1988–93. 10.1073/pnas.1610630114 28167780 PMC5338447

[74] Zhang X Lepelley A Azria E Lebon P Roguet G Schwartz O. Neonatal plasmacytoid dendritic cells. (pDCs) display subset variation but can elicit potent anti-viral innate responses. PLoS ONE. (2013) 8:e52003. 10.1371/journal.pone.0052003 23326320 PMC3542339

[75] Cella M Facchetti F Lanzavecchia A Colonna M. Plasmacytoid dendritic cells activated by influenza virus and CD40L drive a potent TH1 polarization. Nat Immunol. (2000) 1:305–10. 10.1038/79747 11017101

[76] Grouard G Rissoan MC Filgueira L Durand I Banchereau J Liu YJ. The enigmatic plasmacytoid T cells develop into dendritic cells with interleukin. (IL)-3 and CD40-ligand. J Exp Med. (1997) 185:1101–11. 10.1084/jem.185.6.1101 9091583 PMC2196227

[77] Hoeffel G Ripoche AC Matheoud D Nascimbeni M Escriou N Lebon P. Antigen crosspresentation by human plasmacytoid dendritic cells. Immunity. (2007) 27:481–92. 10.1016/j.immuni.2007.07.021 17869134

[78] Alculumbre SG Saint-André V Di Domizio J Vargas P Sirven P Bost P. Diversification of human plasmacytoid predendritic cells in response to a single stimulus. Nat Immunol. (2017) 19:63–75. 10.1038/s41590-017-0012-z 29203862

[79] Macal M Jo Y Dallari S Chang AY Dai J Swaminathan S. Self-renewal and toll-like receptor signaling sustain exhausted plasmacytoid dendritic cells during chronic viral infection. Immunity. (2018) 48:730–44 e5. 10.1016/j.immuni.2018.03.020 29669251 PMC5937984

[80] Stuart T Satija R. Integrative single-cell analysis. Nat Rev Genet. (2019) 20:257–72. 10.1038/s41576-019-0093-7 30696980

